# The impact of body mass index on the diagnostic and surgical outcomes in primary hyperparathyroidism

**DOI:** 10.1590/1806-9282.20240989

**Published:** 2025-05-02

**Authors:** Nazim Serhat Parlak, Süleyman Çağlar Ertekin, Turkay Kırdak

**Affiliations:** 1 Uludag University, Department of General Surgery – Bursa, Turkey.; 2 Altinbas University, Department of General Surgery – İstanbul, Turkey.

**Keywords:** Body mass index, Obesity, Hyperparathyroidism, Parathyroidectomy

## Abstract

**OBJECTIVE:**

The aim of this study was to investigate the influence of body mass index on the diagnostic and surgical outcomes in patients undergoing parathyroidectomy for primary hyperparathyroidism.

**METHODS:**

A total of 446 patients with primary hyperparathyroidism were divided into four groups according to their body mass index: normal weight (body mass index<25 kg/m^2^) (n=130), overweight (25≤body mass index<30 kg/m^2^) (n=166), obese (30≤body mass index<35 kg/m^2^) (n=112), and morbidly obese (body mass index≥35 kg/m^2^) (n=38). Perioperative findings were compared between the groups.

**RESULTS:**

The preoperative median parathormone level in the morbidly obese group (204 pg/mL, min:max 72:1,178) was significantly lower than that in the normal-weight (246 pg/mL, min:max 60:4,262) (p=0.026) and obese (251 pg/mL, min:max 74:2,094) (p=0.012) groups. The osteoporosis rate in the normal-weight group (51%) was higher than that in the overweight (35.4%) (p=0.041) and morbidly obese (25%) (p=0.023) groups. The symptomatic hypocalcemia rate in the normal-weight group (10.2%) was significantly higher than that in the obese group (1.8%) (p=0.017).

**CONCLUSION:**

Normal-weight patients with primary hyperparathyroidism have higher blood parathormone values, higher rates of osteoporosis, and postoperative symptomatic hypocalcemia compared to patients with higher body mass index. For this reason, the surgeon should consider the possibility of symptomatic hypocalcemia after undergoing parathyroidectomy for primary hyperparathyroidism in normal-weight cases.

## INTRODUCTION

Worldwide, the incidence of obesity is increasing daily^1^. Consequently, the impact of body mass index (BMI) on the diagnostic and therapeutic outcomes of surgical diseases, including primary hyperparathyroidism (PHPT), has been extensively studied^2–6^. Studies show that patients with PHPT tend to have higher BMI compared to eucalcemic controls, and BMI has increased over time among these patients^5,6^. Limited studies on the effect of BMI on PHPT outcomes indicate no difference in blood Ca^+2^ levels between groups, but higher BMI correlates with elevated preoperative parathormone (PTH) levels and larger adenomas^5,7–9^. Additionally, osteoporosis is less common in obese patients compared to those with normal weight^8,9^. The clinical presentation of PHPT can be influenced by factors, such as vitamin D levels, age, genetics, ethnicity, and regional differences^10–17^. This study aims to investigate the effect of BMI on the perioperative findings in patients with PHPT.

## METHODS

Cases consecutively operated due to PHPT between 2005 and 2015 were retrospectively investigated. The exclusion criteria were secondary and tertiary hyperparathyroidism (HPT), multiple endocrine neoplasia syndrome, previous parathyroidectomy or thyroidectomy, creatinine>1.4 mg/dL, and insufficient data on BMI. A total of 446 consecutive patients were included in the study. The patients were divided into four groups according to their BMI: normal weight (BMI<25 kg/m^2^) (n=130), overweight (25≤BMI<30 kg/m^2^) (n=166), obese (30≤BMI<35 kg/m^2^) (n=112), and morbidly obese (BMI≥35 kg/m^2^) (n=38). Eleven cases had a BMI <18 kg/m^2^ These cases were included in the normal-weight group.

Preoperative laboratory findings such as PTH, Ca^+2^, 25-OH-vitamin D, bone mineral density (BMD), ultrasonography (USG) and scintigraphy findings, intraoperative findings, and postoperative findings were compared between the groups. This was a retrospective review of a prospectively collected parathyroid database.

The results obtained from the dual-energy X-ray absorptiometry (DXA) measurements were defined as follows: normal: a T-score of −1 standard deviation (SD) or above, osteopenia: a T-score between −1.0 and −2.5 SD, and osteoporosis: a T-score of −2.5 SD or below.

The result of the imaging method was accepted as "successful" if the abnormal parathyroid gland(s) was (were) localized via USG or scintigraphy and if the outcomes were concordant with the operational findings.

The operations were performed by two surgeons experienced in the endocrine surgery field. The number of concomitant thyroidectomies, the number of abnormal parathyroid glands observed, and the adenoma sizes were compared when evaluating the intraoperative findings. The inability to detect the abnormal parathyroid gland during the surgery was accepted as a negative exploration. Parathyroid tumor dimension was determined as the longest dimension measured during the surgery. If there were more than one enlarged parathyroid gland, the larger one was used for comparison. Intraoperative PTH was routinely measured. The first measurement was performed just prior to the incision, and the second measurement was performed 10 min after the excision of the adenoma. The first PTH level was accepted as the basal value, and the percentage change (PC) in the PTH value in the second measurement, compared to the first, was compared between the groups.

The preoperative laboratory values of the patients were those measured 1 month before the surgery. When more than one laboratory value such as Ca^+2^ or PTH was documented preoperatively, the highest level was used for comparison. The postoperative PTH and Ca^+2^ values were those measured within the postoperative first 12–24 h. The normal limits in laboratory measurements were accepted as follows: PTH: 15–68 pg/mL, total Ca^+2^: 8.4–10.2 mg/dL, urinary Ca^+2^: 100–300 mg/24 h, alkaline phosphatase: 40–150 U/L, and 25-OH-vitamin D: 15–100 ng/mL.

When evaluating the postoperative complications, symptomatic cases were accepted as hypocalcemic, cases to be re-operated due to hematomas were accepted as cases with bleeding, and cases with voice loss were accepted as those with recurrent laryngeal nerve palsy. Local ethical committee approval was obtained for the study.

The IBM SPSS Statistics 22 program was used for statistical analysis. The suitability of the parameters to normal distribution was evaluated using the Shapiro-Wilk test. In addition to the descriptive statistic methods (mean, standard error, and frequency), the one-way analysis of variance test was used for the comparison of the quantitative data of the normally distributed parameters, and Tukey's honestly significant difference test was used for the detection of the group responsible for the difference. The Kruskal-Wallis test was used for the intergroup comparison of the non-normally distributed parameters. The Mann-Whitney U test was used for the comparison of two non-normally distributed parameters. The chi-square test, Fisher's exact test, and continuity (Yates) correction were used for the comparison of the qualitative data. Statistical significance was accepted as p<0.05.

## RESULTS

PHPT cases were distributed as follows: normal weight (n=130, 29.1%), overweight (n=166, 37.2%), obese (n=112, 25.1%), and morbidly obese (n=38, 8.5%). Statiscally significant differences in mean BMI (p=0.001) and ages were observed, with the normal-weight group being younger than the obese (p=0.002) and morbidly obese (p=0.023) groups. No statistically significant difference was observed between the other groups with regard to the mean ages (p>0.05). Gender distribution differed significantly (p=0.044), with fewer females in the normal-weight group compared to the obese (p=0.028) and morbidly obese (p=0.032) groups. No statistically significant difference was observed between the remaining groups (p>0.05). The preoperative blood PTH levels varied significantly (p=0.046), with the lowest levels in the morbidly obese group, significantly lower than in the normal-weight (p=0.026) and obese (p=0.012) groups. No statistically significant differences were found in the preoperative blood Ca^+2^, vitamin D, alkaline phosphatase, and 24-h urine Ca^+2^ levels (p>0.05) (Table 1).

**Table 1:** Demographic and preoperative laboratory data of the groups.

	BMI<25	25≤BMI<30	30≤BMI<35	BMI≥35	p
	n=130	n=166	n=112	n=38	
BMI (kg/m^2^)	22.2±0.19	27.4±0.11	32.2±0.13	38.1±0.48	**0.001**
Age (years)	49.4±1.35	53±0.9	55.1±0.91	55.8±1.32	**0.001**
Gender (male/female)	26/104	22/144	11/101	2/36	**0.044**
PTH (pg/mL)	246 (60:4,262)	230 (71:3,000)	251 (74:2,094)	204 (72:1,178)	**0.046**
Ca^+2^ (mg/dL)	11.2±0.1	11.1±0.07	11.2±0.1	11±0.15	0.329
25(OH)D (ng/L)	13.9 (4:338)	14.8 (2.8:68.9)	11.8 (4:47)	13.8 (5.2:34)	0.421
Ur.Ca^+2^ (mg/day)	314.2 (5.7:2,033)	332.5 (9:4,074)	300.7 (5.3:6,000)	174 (4.3:833)	0.098
ALP (U/L)	107 (28:1,665)	100 (10:416)	116 (18:701)	103.5 (63:117)	0.815

Data are presented as mean±SE, median (min:max). BMI: body mass index; PTH: parathormone; Ca^+2^: calcium; Ur.Ca^+2^: 24-h urine calcium; ALP: alkaline phosphatase; 25(OH)D: 25-OH-Vitamin D; SE: standard error. Statistically significant values are denoted in bold.

DXA results for 358 cases showed statistically significant differences (p=0.043), with the morbidly obese group having the highest number of patients with normal BMD. Osteoporosis rates were higher in the normal-weight group compared to the overweight (p=0.041) and morbidly obese (p=0.023) groups. No statistically significant difference was observed between the other groups with regard to the DXA results (p>0.05) (Table 2).

**Table 2:** Dual-energy X-ray absorptiometry findings of the groups.

	BMI<25	25≤BMI<30	30≤BMI<35	BMI≥35	P
	n=102	n=130	n=91	n=32	
Normal	12 (11.8)	15 (11.5)	13 (14.2)	8 (25)	**0.043**
Osteopenia	38 (37.2)	69 (53.1)	38 (41.8)	16 (50)	
Osteoporosis	52 (51)	46 (35.4)	40 (44)	8 (25)	
Lomber-T	-2.2 (-5.4:3.4)	-2 (-5.1:3.7)	-2 (-4.7:2.8)	-1.8 (-5.2:1.6)	0.249
Lomber-Z	-1.4 (-4.8:3.4)	-1.1 (-4.5:4.2)	-1 (-3.8:3.8)	-0.9 (-4.4:4)	**0.043**
Femur-T	-1.6 (-3.9:2.1)	-1.3 (-3.2:1.9)	-1.3 (-6.3:1.9)	-0.2 (-3:2)	**0.001**
Femur-Z	-0.9 (-3.6:2.2)	-0.3 (-3:4.2)	-0.4 (-5.5:2.3)	0.6 (-2.6:3)	**0.001**

Data were presented as n (%) and median (min:max). BMI: body mass index. Statistically significant values are denoted in bold.

No statistically significant differences were found in USG and scintigraphy pre-detection rates between the groups (p>0.05). Additionally, there were no statistically significant differences in intraoperative parameters, adenoma size, operation duration, concomitant thyroidectomy rates, or intraoperative PTH measurements (p>0.05). The postoperative PTH levels at 12–24 h showed no statistically significant differences (p>0.05). However, the normal-weight group had a higher reduction in Ca^+2^ levels compared to the obese group (p=0.024). Transient hypocalcemia was more common in the normal-weight group compared to the obese group (p=0.017). No statistically significant differences were found in transient hypocalcemia rates, postoperative bleeding, recurrent laryngeal nerve palsy, or hospital stay duration among the remaining groups (p>0.05) (Table 3).

**Table 3:** Success of preoperative imaging studies in identifying pathologic parathyroid and intra- and postoperative features belong to groups.

	BMI<25	25≤BMI<30	30≤BMI<35	BMI≥35	p
	n=130	n=166	n=112	n=38	
USG	114 (87.6)	138 (83.1)	96 (85.7)	33 (86.8)	0.743
Scintigraphy	115 (88.4)	145 (87.3)	103 (91.9)	31 (81.6)	0.351
Single adenoma	114 (87.7)	137 (82.5)	100 (89.3)	34 (89.5)	0.375
Double adenomas	6 (4.6)	19 (11.4)	10 (8.9)	2 (5.3)	
Triple adenomas	3 (2.3)	4 (2.4)	1 (0.9)	1 (2.6)	
Negative exploration	7 (5.4)	6 (3.6)	1 (0.9)	1 (2.6)	0.280
IoPTH_PC_	-82 (-98: −15)	-80 (-99: −44)	-81 (-98: −16)	-79 (-97: −5)	0.980
Dimension^a^ (mm)	20 (6:60)	20 (5:50)	20 (7:60)	19.5 (8:46)	0.363
Thyroidectomy^b^	23 (18.1)	35 (21.7)	20 (18.9)	11 (29.7)	0.443
Duration^c^ (min)	60 (20:200)	65.5 (20:210)	60 (21:200)	75 (35:180)	0.272
PTH_po12–24 h_ (pg/mL)	21.5 (1.4:810)	25.8 (1.6:558)	26.2 (2:264.7)	21.3 (3:282.4)	0.437
Ca^+2^_po12–24 h_ (mg/dL)	8.6±0.10	8.6±0.07	9.3±0.08	9.3±0.15	0.077
Ca^+2^_PC_	-23.5±0.76	-21.5±0.67	-20.4±0.64	-20.2±1.5	**0.021**
Bleeding	1 (0.8)	0 (0)	1 (0.9)	0 (0)	0.635
Hypocalcemia^d^	3 (10.2)	9 (5.5)	2 (1.8)	1 (2.7)	**0.036**
RLN palsy	1 (0.8)	1 (0.6)	4 (3.6)	0 (0)	0.129
Hosp. stay (day)	1 (1:8)	1 (0:18)	1 (0:6)	1 (1:2)	0.136

Data were presented as n (%) and median (min:max) and mean±SE.

a Adenoma dimension;

b Concomitant thyroidectomy;

c Duration of surgery;

d Symptomatic hypocalcemia.

Hosp: hospital; Hematoma: bleeding requiring reoperation; Hypocalcemia: transient symptomatic hypocalcemia; IoPTH _PC_: percent change of intraoperative parathormone between preexcision and postexcision measurements; PTH_po12–24 h_: parathormone values measured within the postoperative first 12–24 h; Ca^+2^_po12–24 h_: calcium values measured within the postoperative first 12–24 h; RLN: recurrent laryngeal nerve; Ca^+2^_PC_: percent change of total blood calcium between preoperative and postoperative first day; BMI: body mass index; USG: ultrasonography. Statistically significant values are denoted in bold.

## DISCUSSION

The number of studies investigating the effect of BMI on PHPT in the literature is limited. Although these studies report no difference between groups with regard to blood Ca^+2^ levels, patients with higher BMI have been reported to have higher preoperative blood PTH levels and larger parathyroid adenomas^5,7–9^. However, there are some important differences between these studies and our study with regard to the characteristics of the BMI groups. In some of these studies, the BMI groups were observed to be similar with regard to age and gender^5,8,9^. However, in the study of Pitt et al.^7^ and our study, the BMI groups were different with regard to age and gender. Pitt et al. reported that morbidly obese cases were younger than normal-weight cases and that the normal-weight group included more women than men. In contrast, patients in the normal-weight group were younger than the patients in the morbidly obese group and the rate of women was lower in our study. Furthermore, the mean age of all participants revealed a younger value among patients with PHPT in our study (mean age: 52 years) compared to that in other studies.

On the other hand, comparison of the groups with the highest and lowest BMI values revealed a significantly higher mean preoperative blood PTH level in the high BMI group in the present studies^5,7–9^. In contrast, the blood PTH levels in the normal-weight group were significantly higher than those in the morbidly obese group in our study.

There may be several reasons for the above-mentioned differences in the age and blood PTH of the patients between our study and other studies. As known, the clinical presentation of PHPT is more serious among young individuals^10,11,12^. On the other hand, the PTH levels were observed to be higher in the morbidly obese group in Pitt et al.'s study^7^, whereas they were higher in the normal-weight group in our study, which may seem like different outcomes at the beginning. However, the common aspect of the morbidly obese group in Pitt et al.'s study and the normal-weight group in our study was that they were the youngest groups in both studies. As mentioned above, the PTH levels were higher in younger patients. Thus, the higherPTH levels and the heavier glands observed in the morbidly obese group in the study of Pitt et al. may be due to the younger age of the group compared to other BMI groups.

Another factor that leads to the severity of the clinical presentation of PHPT is vitamin D insufficiency or deficiency^10^. A comparison of the present studies revealed that the 25-OH-vitamin D levels of the patients in our study (both of the groups with the lowest and highest BMI) were lower than those observed in the BMI groups of other studies. Younger age, higher blood PTH levels, and lower vitamin D levels observed in the normal-weight cases in our study are not compatible with the studies investigating the relationship between PHPT and BMI but in compliance with the literature on classical symptomatic PHPT.

There is not much data about BMD in the studies investigating the relationship between PTH and BMI. Tran^8^ and Adam^9^ observed a higher osteoporosis rate in normal-weight cases compared to obese patients. The outcomes obtained in our study were similar. Moreover, the tendency toward a reduction in the PC rate of postoperative blood Ca^+2^ level and the incidence of symptomatic hypocalcemia were found to be higher in the normal-weight cases compared to the obese patients in our study. This condition may be related to hungry bone syndrome, which occurs after parathyroid surgery due to the sudden uptake of minerals by the overactive bones, leading to a rapid drop in blood Ca^+2^ levels, particularly in patients with bone disease associated with long-standing HPT^13^. Besides, these findings were in compliance with the outcome that "the clinical presentation of PHPT is more serious among normal-weight individuals," which was determined in our study. However, the absence of a difference between the mean postoperative Ca^+2^ levels of the BMI groups and the partial subjectivity of symptomatic hypocalcemia partly support our conclusion.

Another reason for the differences in the findings may be the different frequency and severity of obesity in different geographical regions^1^. For example, the rate of high-BMI cases is lower in our study than that in other studies. In addition to these reasons, it should be considered that the clinical presentation of PHPT may be affected by genetic, ethnic, economic, cultural, and climatic factors^14–17^. Depending on these factors, there may be differences between the cases with PHPT living in different geographical regions with regard to parameters, such as age, bone findings, blood PTH and vitamin D levels, and the adenoma size. However, the present studies in the literature investigating the relationship between PHPT and BMI are of USA origin, and no sufficient data is available for the other regions^5,7–9^. In our study, conducted in a region other than the USA, findings such as younger age, higher PTH levels, and lower vitamin D levels observed in our cases may indicate a more severe clinical course of PHPT. However, the asymptomatic PHPT rates in the USA are very high. Thus, the outcomes of this study suggest that the effect of age, vitamin D levels, and other factors leading to regional differences on the clinical presentation of PHPT is more than the effect of obesity.

## CONCLUSION

We found that normal-weight patients with PHPT have higher blood PTH values, higher rates of osteoporosis, and postoperative symptomatic hypocalcemia compared to patients with higher BMI. For this reason, the surgeon should consider the possibility of symptomatic hypocalcemia after undergoing parathyroidectomy for PHPT in normal-weight cases. In addition to this, the results of this study suggest that the effect of factors such as age and vitamin D level and other possible factors that may lead to regional differences in the clinical presentation of PHPT is more than the effect of obesity.

## References

[1] Wong MCS, Huang J, Wang J, Chan PSF, Lok V, Chen X (2020). Global, regional and time-trend prevalence of central obesity: a systematic review and meta-analysis of 13.2 million subjects. Eur J Epidemiol.

[2] Runkel M, Diallo TD, Lang SA, Bamberg F, Benndorf M, Fichtner-Feigl S (2021). The role of visceral obesity, sarcopenia and sarcopenic obesity on surgical outcomes after liver resections for colorectal metastases. World J Surg.

[3] Khachfe HH, Hammad AY, AlMasri S, deSilva A, Kraftician J, Lee KK (2023). Obesity is associated with increased risk for adverse postoperative outcomes after distal pancreatectomy for pancreatic ductal adenocarcinoma. J Surg Res.

[4] Konishi T, Fujiogi M, Michihata N, Niwa T, Morita K, Matsui H (2022). Impact of body mass index on short-term outcomes after differentiated thyroid cancer surgery: a nationwide inpatient database study in Japan. Eur Thyroid J.

[5] Lin X, Fan Y, Zhang Z, Yue H (2021). Clinical characteristics of primary hyperparathyroidism: 15-year experience of 457 patients in a single center in China. Front Endocrinol (Lausanne).

[6] Pal R, Arya AK, Aggarwal A, Singh P, Dahiya D, Sood A (2020). Weight gain after curative parathyroidectomy predicts increase in bone mineral density in patients with symptomatic primary hyperparathyroidism. Clin Endocrinol (Oxf).

[7] Pitt SC, Panneerselvan R, Sippel RS, Chen H (2010). Influence of morbid obesity on parathyroidectomy outcomes in primary hyperparathyroidism. Am J Surg.

[8] Tran H, Grange JS, Adams-Huet B, Nwariaku FE, Rabaglia JL, Woodruff SL (2014). The impact of obesity on the presentation of primary hyperparathyroidism. J Clin Endocrinol Metab.

[9] Adam MA, Untch BR, Danko ME, Stinnett S, Dixit D, Koh J (2010). Severe obesity is associated with symptomatic presentation, higher parathyroid hormone levels, and increased gland weight in primary hyperparathyroidism. J Clin Endocrinol Metab.

[10] Rao SD, Miragaya J, Parikh N, Honasoge M, Springer K, Harn M (2020). Effect of vitamin D nutrition on disease indices in patients with primary hyperparathyroidism. J Steroid Biochem Mol Biol.

[11] El-Hajj Fuleihan G, Chakhtoura M, Cipriani C, Eastell R, Karonova T, Liu JM (2022). Classical and nonclassical manifestations of primary hyperparathyroidism. J Bone Miner Res.

[12] Kim SJ, Shoback DM (2021). Sporadic primary hyperparathyroidism. Endocrinol Metab Clin North Am.

[13] Carsote M, Nistor C (2023). Forestalling hungry bone syndrome after parathyroidectomy in patients with primary and renal hyperparathyroidism. Diagnostics (Basel).

[14] Kandil E, Tsai HL, Somervell H, Dackiw AP, Tufano RP, Tufaro AP (2008). African Americans present with more severe primary hyperparathyroidism than non-African Americans. Surgery.

[15] Moosgaard B, Vestergaard P, Heickendorff L, Melsen F, Christiansen P, Mosekilde L (2005). Vitamin D status, seasonal variations, parathyroid adenoma weight and bone mineral density in primary hyperparathyroidism. Clin Endocrinol (Oxf).

[16] Mercanoglu Efe E, Kirdak T, Aykut G, Kaya Argadal E, Kaya E, Ersoy C (2022). Comparison of the effect of anesthetic agents on blood levels of parathyroid hormone and ionized calcium: a prospective randomized controlled trial. Int J Clin Pract.

[17] Broekhuis JM, Cote MP, Collins RA, Gomez-Mayorga JL, Chaves N, James BC (2024). Association of patient-practitioner sex concordance with specialist referral in primary hyperparathyroidism. Surgery.

